# Physiological and Clinical Impact of Repeated Inhaled Oxygen Variation on Erythropoietin Levels in Patients After Surgery

**DOI:** 10.3389/fphys.2021.744074

**Published:** 2021-09-27

**Authors:** Maher Khalife, Mohammed Ben Aziz, Costantino Balestra, Joseph Valsamis, Maurice Sosnowski

**Affiliations:** ^1^Department of Anesthesiology, Institut Jules Bordet, Université Libre de Bruxelles (ULB), Brussels, Belgium; ^2^Environmental and Occupational Physiology Laboratory, Haute Ecole Bruxelles-Brabant, Brussels, Belgium; ^3^Universitair Verplegingscentrum (UVC) Brugmann Site Victor Horta, Clinical Biology, Brussels, Belgium

**Keywords:** hematopoiesis, normobaric hyperoxia, normobaric oxygen paradox (NOP), transfusion alternatives, relative hypoxia

## Abstract

The “Normobaric Oxygen Paradox” (NOP) is a physiologic mechanism that induces an increase of endogenous erythropoietin (EPO) production by creating a state of relative hypoxia in subjects previously exposed to hyperoxia, followed by a rapid return to normoxia. Oxygen exposure duration and inspired oxygen fraction required to observe a significant increase in EPO or hemoglobin are not clearly defined. Consequently, we here study the effect of one model of relative hypoxia on EPO, reticulocytes and hemoglobin stimulation in patients after surgery. Patients were prospectively randomized in two groups. The O_2_ group (*n* = 10) received 100% oxygen for 1 h per day for eight consecutive days, via a non-rebreathing mask. The control group (*n* = 12) received no oxygen variation. Serum EPO, hemoglobin and reticulocyte count were measured on admission and postoperatively on days seven and nine. Percentage EPO at day nine with respect to the baseline value was significantly elevated within the groups [O_2_ group: 323.7 (SD ± 139.0); control group: 365.6 (SD± 162.0)] but not between them. No significant difference was found between the groups in terms of reticulocytes count and hemoglobin. Our NOP model showed no difference on EPO increase between the two groups. However, both groups expressed separately significant EPO elevation.

## Introduction

At the turn of the 21st century, clinicians were optimistic that an alternative to blood transfusion would be developed. This pressing need arose mainly from three factors: a high demand on limited blood supplies, the reluctance of patients to consume blood components and the risk associated with blood transfusion (Vossoughi et al., 2018). Though the best “alternative” to blood transfusions is to prevent and avoid them, this is not always possible. The development of “artificial blood” or a “blood substitute” is under investigation (Azuma et al., 2017; Davis et al., 2018), but there is still no optimal substitute for human blood although other alternatives have been developed (Bursi et al., 2009; Ruhl et al., 2009).

The use of the pharmacological stimulating agent, erythropoietin (EPO), has proven its efficacy in the treatment of chronic anemia. EPO induces red blood cell production by activating red bone marrow progenitor cells, which in turn stimulates reticulocytes (Jelkmann and Jelkmann, 2013). However, several side effects have been reported on its pharmacological administration (Bohlius et al., 2009). Moreover, the price of such medication remains high and its availability for patients in certain countries is limited.

A few years ago, a phenomenon known as the “normobaric oxygen paradox” (NOP) was described. This technique, whereby a high concentration of oxygen (O_2_) is given to spontaneously breathing subjects at normobaric pressure, increase significantly the production of endogenous erythropoietin (Balestra et al., 2006, 2010). The mechanism explaining this phenomenon is based on a cellular model adjusted to hypoxia and it depends on the availability of the reactive oxygen species (ROS). The principle element is the complex formed by the hypoxia-inducible factors 2 alpha and 1 alpha (HIF-1α and 2α) and the tumor-suppressing Von Hippel Lindau protein (VHLp), which is constantly bound to ubiquitin ligase (Ivan et al., 2001; Jaakkola et al., 2001). A limited availability or absence of ROS leads to the complex dissociation and dimerization of HIF-1α with HIF-1β. This latter complex induces EPO gene expression and EPO hormone production (Masson et al., 2001).

Much of the literature suggests that the NOP effect could lead to the production of endogenous EPO (Balestra et al., 2006; Burk, 2007; Ciccarella et al., 2011; Cimino et al., 2012). Recently, a clinical study and limited to 48 h has supported the evidence of the NOP effect (Donati et al., 2017). Conversely, Keramidas et al. (2011, 2012) suggest that the NOP effect is not always clear cut. However, it is thought that the specific period of hyperoxia and oxygen concentration is key to induce the effect. Consequently, it might be possible that the proposed regimen was not optimal to induce the NOP effect. Unfortunately, the precise regime need is still unknown.

The present study evaluates whether the application of one NOP regime could efficiently increase EPO level on patients undergoing deep inferior epigastric perforator flap surgery (DIEP).

## Materials and Methods

Inclusion criteria of this prospective single-center, controlled, randomized study are female patients of a minimum 18 years old eligible for DIEP surgery. Exclusion criteria included: severe renal insufficiency (GFR < 60 ml/min and creatinine > 2 mg/dl), bleeding necessitating iterative transfusion per and/or postoperatively, severe respiratory syndrome requiring constant oxygen administration and an intolerance to wearing an oxygen mask. All samples are collected and processed at the Institut Jules Bordet, Belgium, with the exception of EPO serum, which is sent to the clinical laboratory of CHU-Brugmann.

A total of twenty six patients scheduled for plastic surgery were enrolled between April and November 2017. The study was approved by the local ethics committee of Institut Jules Bordet, Belgium (approval number CE2103). The work was in accordance to the declaration of Helsinki. Written informed consent was obtained from all participants.

Patients are randomized to two parallel groups at the end of the surgery. Randomization is performed using a Microsoft Excel program. The knowledge of the treatment randomization is open to all except for the laboratory staff.

In our study, sample size calculations are not performed, however the sample size is equal or greater to previously published studies (Ciccarella et al., 2011; Cimino et al., 2012).

All patients underwent general anesthesia. During the surgery, oxygen administration was maintained between 40 and 50%.

All patients were free from mechanical ventilation at the end of the surgery and were admitted to the intensive care unit (ICU) for 48 h. During this ICU stay, a baseline oxygen was administered to every patient via a cannula to ensure saturation above 98%. Oxygen variation started on the first day postoperatively between eight and 10 o'clock in the morning. The first group (O_2_ group) received 100% oxygen for 1 h per day for eight consecutive days post-surgery, via a non-rebreathing mask. The second group (control group) received no oxygen variation during the postoperative period. All patients received a continuous dose (1 or 2 liters/min.) of Oxygen to reach 98% of saturation for 48 h postoperatively during ICU stay. After ICU discharge, no oxygen administration was necessary in either control or O_2_ group since 98% of saturation was reached with spontaneous air breathing. However, clinicals signs and surgical complications evolution were constantly evaluated by the staff involved in the study.

The transfusion threshold was set to hemoglobin 9 g/dl along with clinical signs.

Clinical information collected on each patient included age and body mass index.

Clinical data collected included the times of start and end surgery, blood loss and duration of hospital stay. EPO, hemoglobin, hematocrit, reticulocyte count where recorded. Other laboratory data included platelet count, urea, creatinine, ferritin, transferrin saturation, iron, prothrombin time and activated prothrombin time.

To measure EPO baseline values, venous blood samples were collected at eigh am before exposure to peroperative hyperoxia. Follow up lab samples were collected on day two, three, four and nine postoperatively. EPO analysis was performed using an IMMULITE^®^ 2000 (Siemens), with chemiluminescence. The occurrence of any postoperative complications was also recorded. All data were introduced in a Microsoft office program coming from the hospital electronic medical program. The risk of bias was not assessed at the time of the study.

### Study Outcomes

The primary end point of the study was to compare the effects of the NOP by evaluating the EPO percentage mean change from baseline to day nine within and between the two groups. The secondary end points included the comparison of the reticulocyte count, hemoglobin level measured at baseline and day nine as well as any complications could occur.

### Statistical Analysis

Standard statistical analyses were performed, including mean and standard deviation for each treatment group. A one-sample paired student *t*-test was used to detect the between- and within-subject treatment difference. Kolmogorov Smirnov tests were performed to assess the normality of the data. Student *t*-test was used for data of patients characteristics, laboratory data as well as the EPO absolute results. Taking the initial value as 100%, percentage changes in EPO were calculated, thereby observing relative changes rather than the absolute values. The raw data are available from the Journal office. An ANOVA test for repeated measures was also applied for the percentage changes in EPO at different days, with Geisser-Greenhouse's epsilon correction for standard deviations. Multiple comparisons were performed by means of Tukey's test. Statistical significance was set at *p* < 0.05.

## Results

Twenty-six patients were enrolled in the study. Only twenty-two patients were included in the final analysis; one set of data was missing from the O_2_ group and three patients (two in the O_2_ and one in the control group) required a transfusion during their hospital stay and so were withdrawn from the study. The clinical characteristics of the two groups are shown in Table 1A. With the exception of age, we did not notice any significant difference between the groups.

**TABLE 1A:** Patient characteristics for the O_2_ and control groups.

**Variable**	**O_**2**_ group (*n* = 10)**	**Control group (*n* = 12)**	***P***
Age (years)	57.70 ± 8.38	49.25 ± 9.23	0.037
BMI^#^ (Kgm^−2^)	27.06 ± 5.03	24.87 ± 4.26	NS^‡^
Duration of surgery (min^§^)	654.5 ± 52.6	647.4 ± 56.6	NS
Hospital stay (days)	11.60 ± 2.59	10.00 ± 2.17	NS
Blood loss (ml)	1,082 ± 285	1,160 ± 555	NS

Data given as mean ± SD^*^.

*Standard deviation.

# *Body mass index*.

‡ *Not significant*.

§ *Minutes*.

Also we didn't record significant difference comparing the baseline for the platelet count, urea, creatinine, ferritin, transferrin saturation, iron, prothrombin time and activated prothrombin time to their ninth day values (Table 1B).

**TABLE 1B:** Patient laboratory data for the O_2_ and control groups.

**Variable**	**Day**	**O2 group (*N* = 10)**	**Control group (*N* = 12)**	***P***
Platelet (10^9^L-1)	D0	221 ± 38	267 ± 51	NS^‡^
	D9	346 ± 88	439 ± 149	NS
Urea (mg.dl-1)	D0	34.70 ± 7.21	28.33 ± 9.69	NS
	D9	20.60 ± 5.92	20.25 ± 6.20	NS
Creatinine (mg.dl-1)	D0	0.85 ± 0.11	0.80 ± 0.13	NS
	D9	0.67 ± 0,08	0.61 ± 0.12	NS
Ferritin (g.dl-1)	D0	136.4 ± 79.4	77.4 ± 56.9	NS
	D9	216.4 ± 91.9	194.2 ± 124.3	NS
Transferrin saturation (%)	D0	30 ± 11	33 ± 13	NS
	D9	16 ± 6	14 ± 5	NS
Iron (g.dl-1)	D0	98.9 ± 32.3	117.8 ± 36.1	NS
	D9	48.5 ± 18.7	46.0 ± 23.1	NS
PTT^§^ (%)	D0	103.2 ± 12.7	103.1 ± 6.1	NS
	D9	86.0 ± 7.6	83.7 ± 6.6	NS
aPTT^#^ (sec)	D0	25.3 ± 3.1	25.0 ± 1.9	NS
	D9	25.8 ± 3.1	25.3 ± 2.5	NS

Data given as mean ± SD^*^.

*Standard deviation.

‡ *Not significant*.

§ *Prothrombin time*.

# *Activated prothrombin time*.

*D0, baseline; D9, day 9*.

In absolute values, we noticed more EPO production in the control group than in the O_2_ group with a peak level at the third day postoperatively (Table 2).

**TABLE 2:** Evolution over time of EPO in O_2_ group and control group expressed as mean ± (SD^*^).

	**Day 0**	**Day 2**	**Day 3**	**Day 4**	**Day 9**
**EPO (mIU/ml)**					
O_2_ group (*n* = 10)	10.38(±5.25)	18.39(±13.3)	28.45(±12.38)	36.33(±12.29)	36.53(±27.21)
Control group (*n* = 12)	9.867(±3.60)	21.99(±9.32)	42.19(±24.07)	39.17(±16.15)	32.15(±8.78)

* *Standard deviation*.

**Figure 1:**
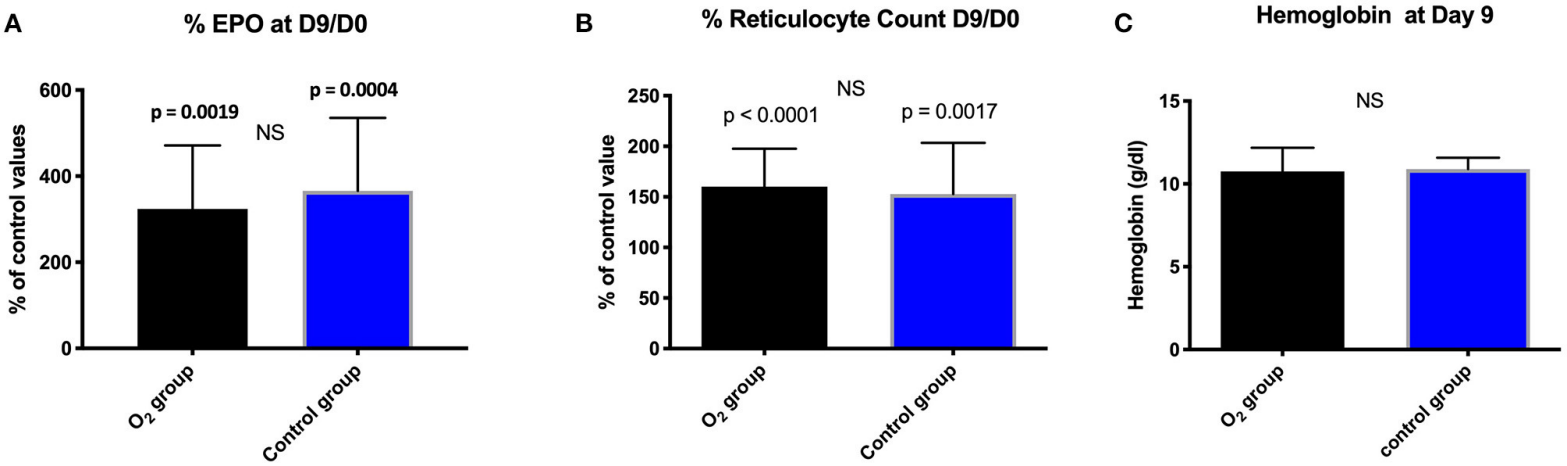
Comparison between and within the groups in percentage: **(A)** EPO; **(B)** Reticulocyte count expressed as mean ± standard deviation at day 9 (D9) with respect to the baseline value (D0); (NS, non-significant). **(C)**: Evolution of hemoglobin (g/dl) expressed as mean ± standard deviation at day 9 in the two groups; (NS, not significant).

A significant increase of EPO production was noted within the groups but not between them (Table 3; Figure 1A).

**TABLE 3:** Percentage EPO expressed as mean (±SD^*^) for each group with respect to baseline value (D0^†^) at different days across the study period (ANOVA test).

**Di^‡^/D0 x100**	**Day 2**	**Day 3**	**Day 4**	**Day 9**
**EPO (%)**				
O_2_ group	192.1 (±112.1)	323.8 (±153.1)	392.9 (±118.8)	323.7 (±139.0)
***P*** **-value**	**0.0288**	**0.0013**	**<0.0001**	**0.0019**
Control group	260.6 (±168.3)	466.8 (±250.3)	448.2 (±250.1)	365.6 (±162.0)
***P*** **-value**	**0.007**	**0.0004**	**0.0005**	**0.0004**

* *Standard deviation*.

‡ *Different days over the study period*.

† *Baseline value*.

*p-values for within-subject change from baseline*.

At Day nine and with respect to the baseline, we did not notice any significant difference between the groups in terms of reticulocytes count and hemoglobin (Figures 1B,C).

The hemoglobin levels at day two postoperatively were, respectively, 9.92 (SD± 1.113) and 9.94 (SD± 0.738) in the O_2_ group and in the control group.

Multiple comparison of the means of percentage of EPO levels at day three, four and nine with respect to day two within each group showed a significant difference, respectively, for O_2_ group: 324 ± 153%; *p* < 0.05, 393 ± 119; *p* < 0.001, 324 ± 139; *p* < 0.05. Control group: 466.8 ± 250; *p* < 0.01, 448.2 ± 250; *p* < 0.05, 365.6 ± 162; *p* < 0.05. The group effects (R square) were, respectively, 0.496 in O_2_ group and 0.362 in the control group.

We did not identify any significant surgical complications between the groups.

## Discussion

The production of EPO between individuals and within individuals varies greatly across the day, which is probably due to each person's circadian rhythm (Balestra and Germonpre, 2012). Given this variation, we choose to use an individual's relative results with respect to their own baseline, rather than absolute values. Since red blood cell transfusion could alter EPO production, we excluded the transfused patients from statistical analysis (Schwarz et al., 2005). Our study was designed to investigate the effect of “relative hypoxia” in a specific NOP model. We noted elevated EPO values in both groups which were significantly different within them but not in between. Although EPO responds in hours post oxygen exposure (Balestra et al., 2006; Ciccarella et al., 2011; Revelli et al., 2013; Kiboub et al., 2018), we intended to go forward in our EPO analysis. The rationale behind this measurement at day nine was to correlate it with the reticulocytes profile since in another clinical setting study by our group, we identified reticulocytes increase (Lafere et al., 2013) and we couldn't measure EPO levels at that time point. Furthermore, administration of NAC alone without oxygen (as opposed to Momeni's study)(Momeni et al., 2011) showed an increase of EPO after 8 days (Zembron-Lacny et al., 2009), therefore this time frame was interesting to be investigated. Contrary to most other studies where EPO is measured on healthy volunteers, in our study, subjects were surgical patients. Consequently, their per and postoperative oxygen intake are determined by clinical needs. In fact, during anesthesia, patients were ventilated with at least 40–50% oxygen concentration. Therefore, both groups were exposed to prolonged oxygen till the end of the surgery.

On the other hand, patients wore nasal cannula delivering a constant flow of oxygen during their 48 h stay in the ICU. Oxygen delivery is part of postoperative routine and is adapted to the patient's needs to achieve 98% pulse oximetric saturation. When designing the study, we did not expect that a small variation could influence EPO stimulation. So, our control group received somehow a mild hyperoxia. In a recently young healthy volunteers study (Fratantonio et al., 2021), authors observed a significant increase, up to 4-fold, in nuclear HIF-1α when mild 1 h hyperoxia (30% oxygen) was applied before return to normoxia which in fact represent a 10% change compared to atmospheric oxygen content. Conversely, when 100% oxygen was applied, nuclear HIF-1α increase was of lesser impact. These results could contribute to the understanding of the increased EPO level in our control group and the absence of significant results with respect to the oxygen group.

Normobaric oxygen, if given too often, might not be effective in stimulating EPO synthesis (De Bels et al., 2011, 2012; Balestra and Germonpre, 2012). A high normobaric oxygen concentration seems to be a limiting factor with regard to increasing nuclear HIF-1α production and is considered to rather induce a shift toward an oxidative stress response (Fratantonio et al., 2021). In a randomized clinical trial enrolling cardiac surgery patients, less consistent results are found when delivering a normobaric oxygen fraction of 100% (Ciccarella et al., 2011) than in those studies utilizing 50% normobaric oxygen. However, the study was limited to 48 h mechanically ventilated patients. Also, Keramidas showed that 100% oxygen is not optimal for healthy volunteers (Keramidas et al., 2011). Furthermore, other authors showed that widening the range of normobaric hyperoxia to normobaric hypoxia suppress EPO stimulation (Debevec et al., 2012).

Anemia induced by blood loss could influence EPO synthesis (Alamo et al., 2016). In our study, EPO synthesis was influenced similarly in term of blood loss since no significant difference was identified. Besides, platelet count, prothrombin time and activated prothrombin time variations were similar in both groups. Moreover, no patient reported menstruations during the experimental period.

Another factor that could have influenced EPO production is aging and renal function (Costa et al., 2014; Panjeta et al., 2015). Although the control group was significantly younger than the O_2_ group, renal function was quite similar and consequently this would have little chance to influence EPO synthesis between the groups.

One more factor is the inflammatory reaction induced by surgery. It is thought to increase the iron regulatory peptide hepcidin (Verga Falzacappa et al., 2007) making iron less available. This will compromise the action of EPO (Pak et al., 2006). In the present study, the impact of the inflammatory reaction was not investigated and iron deficiency at the end of the total hospital stay was similar in both groups.

For these above mentioned reasons and probably for other hidden ones, we did not observe any differences between the groups with respect to both the EPO, reticulocyte and hemoglobin levels. However, in a recent study (Khalife et al., 2018) we used a similar protocol but applied a NOP regime with a lower oxygen gradient to patients undergoing abdominal surgery. We observed a statistically significant increase in reticulocytes in the group exposed to the NOP. Different NOP regime, type of pathology and type of surgery may explain these results. In view of its complexity, we suggest to apply different oxygen concentration in combinations with different oxygen administration intervals in order to explore the phenomenon (Rocco et al., 2014; Fratantonio et al., 2021).

The theory behind the NOP effect mechanism may be understood in two different ways. The first involves glutathione activity, which may be modulated by prolonged hyperoxia thus allowing an EPO rebound, as shown after prolonged hyperoxia during diving (Revelli et al., 2013; Donati et al., 2017). The second explanation depends on the HIF-1alpha increase as proposed by De Bels (De Bels et al., 2011). In this paper, HUVEC cells (human umbilical vein endothelial cells) showed a decrease of HIF-1 alpha expression after 2 h of hyperoxia, reaching 0.59 % of control values, followed, 4 h post hyperoxia, by a reactive increase up to 119.1% and to a 176.6% increase at 6 h post hyperoxia. No absolute hypoxia was applied to the cellular culture lines, so hyperoxia was the only trigger for the increased HIF expression (Cimino et al., 2012). In our study, both mechanisms could be in effect to varying degrees within each group, and this may explain the increase observed in both groups.

In our clinical model, these results lead to debate on several questions: What is the influence of prolonged oxygen administration on EPO stimulation, what is the role of the inflammatory reaction induced by surgery?, is a lower normobaric oxygen concentration or gradient enough for EPO stimulation?, how many repetitions of “relative hypoxia” will produce optimal results?, what time between session will be optimal?

Our study has several limitations. The main one is the small sample size. For clinical purposes, the study is also limited by the constraint normobaric oxygen delivered to both groups. The small size of the sample may of course impact the outcome, nevertheless similar number of subjects were included in other works in clinical setting, even if EPO was not measured (Lafere et al., 2013), we are well aware that the optimal sequence to reach maximal NOP effect is not yet defined, thus a sample to clearly tackle the NOP effect is very difficult to determine particularly after newly published nuclear expression data (Fratantonio et al., 2021). The power calculation according to the percentage outcome in other studies of our group for EPO was already reaching 100% with 12 subjects, if the sample size calculation was on the Hb increase in g/dl (*post-hoc* calculation) we reach a sample of 10 subjects to achieve 80% of power.

The power calculation for the study was difficult to perform since no consensus is reached on the link between EPO level needed to have relevant clinical Hb values (Panjeta et al., 2015). However, neuroprotection and cardioprotection are already described with increased EPO in patients, from exogenous and endogenous origin (Bogoyevitch, 2004). We believe that studies as the one proposed will be useful in the future to fine tune the clinical aspects and determine the future studies design, we have to consider this study as a pilot one. Another limiting factor is our non –anemic population. However, given that both groups showed augmented EPO values during the trial, these limitations could or not be used to argue against any possible influence on the NOP effect. Moreover, very recent data in divers seem to confirm that little PO_2_ variations can induce significant EPO variations (Perovic et al., 2020), in fact: the dives performed were 30 meter depth for 30 min once per week for 5 weeks, this is similar to breath 80% of FiO_2_ for 30 min. The authors consider that, in their setting, plasma volume variations could also be a triggering factor. In our experiment we cannot invoke such an effect since this parameter was stabilized.

## Conclusions

This randomized study failed to demonstrate a significant difference in EPO production between the two groups. However, it can be inferred from the results that EPO stimulation could probably be influenced by factors other than the NOP and seems to be affected by even a small oxygen fluctuation as well as a prolonged oxygen exposure. Further investigations are needed to determine an optimal threshold, concentration and interval of administration in surgical, non-surgical and anemic patients.

## Data Availability Statement

The raw data supporting the conclusions of this article will be made available by the authors, without undue reservation.

## Ethics Statement

The studies involving human participants were reviewed and approved by the local Ethics Committee of Institut Jules Bordet, Belgium (approval number CE2103). The patients/participants provided their written informed consent to participate in this study.

## Author Contributions

MK: protocol designer, collecting results, writing manuscript, data interpretations, and supervisor for the whole project. MB: participation for the running protocol (data discussion and analysis) and oxygen administration. CB: contribution to the design of the protocol, interpretation for the data, and revising the final editing manuscript in all aspects (questions regarding the outcomes correctly investigated). JV: clinical biology analysis. MS: contribution to the design of the protocol, interpretation for the data, and revising the final editing manuscript in all aspects (questions regarding the outcomes and results correctly investigated). All authors contributed to the article and approved the submitted version.

## Conflict of Interest

The authors declare that the research was conducted in the absence of any commercial or financial relationships that could be construed as a potential conflict of interest.

## Publisher's Note

All claims expressed in this article are solely those of the authors and do not necessarily represent those of their affiliated organizations, or those of the publisher, the editors and the reviewers. Any product that may be evaluated in this article, or claim that may be made by its manufacturer, is not guaranteed or endorsed by the publisher.

## Abbreviations

NOP: Normobaric Oxygen Paradox
EPO: Erythropoietin
DIEP: Deep Inferior Epigastric Perforator
O_2_: Oxygen
ROS: reactive oxygen species
HIF-1α: hypoxia-inducible factor 1 alpha
VHLp: Von Hippel Lindau protein
GFR: glomerular filtration rate
ICU: intensive care unit.
